# Postprandial lipid responses to an alpha-linolenic acid-rich oil, olive oil and butter in women: A randomized crossover trial

**DOI:** 10.1186/1476-511X-10-106

**Published:** 2011-06-28

**Authors:** Julia Svensson, Anna Rosenquist, Lena Ohlsson

**Affiliations:** 1 Department of Biotechnology, Lund University, P.O. Box 124, SE-221 00 Lund, Sweden; 2 Department of Clinical Sciences, Laboratory of Gastroenterology and Nutrition, Biomedical Centre B11, Lund University, SE-221 84 Lund, Sweden

## Abstract

**Background:**

Postprandial lipaemia varies with gender and the composition of dietary fat due to the partitioning of fatty acids between beta-oxidation and incorporation into triacylglycerols (TAGs). Increasing evidence highlights the importance of postprandial measurements to evaluate atherogenic risk. Postprandial effects of alpha-linolenic acid (ALA) in women are poorly characterized. We therefore studied the postprandial lipid response of women to an ALA-rich oil in comparison with olive oil and butter, and characterized the fatty acid composition of total lipids, TAGs, and non-esterified fatty acids (NEFAs) in plasma.

**Methods:**

A randomized crossover design (n = 19) was used to compare the postprandial effects of 3 meals containing 35 g fat. Blood samples were collected at regular intervals for 7 h. Statistical analysis was carried out with ANOVA (significant difference = P < 0.05).

**Results:**

No significant difference was seen in incremental area under the curve (iAUC) plasma-TAG between the meals. ALA and oleic acid levels were significantly increased in plasma after ALA-rich oil and olive oil meals, respectively. Palmitic acid was significantly increased in plasma-TAG after the butter meal. The ratios of 18:2 n-6 to18:3 n-3 in plasma-TAGs, three and seven hours after the ALA-rich oil meal, were 1.5 and 2.4, respectively. The corresponding values after the olive oil meal were: 13.8 and 16.9; and after the butter meal: 9.0 and 11.6.

**Conclusions:**

The postprandial p-TAG and NEFA response in healthy pre-menopausal women was not significantly different after the intake of an ALA-rich oil, olive oil and butter. The ALA-rich oil significantly affected different plasma lipid fractions and improved the ratio of n-6 to n-3 fatty acids several hours postprandially.

## Background

Increased postprandial plasma triacylglycerol (TAG) levels have been shown to increase the risk of cardiovascular disease (CVD) in women more than in men [1], and since most of the day is spent in the postprandial state, it is important to characterize the ways in which different dietary fatty acids (FAs) influence postprandial lipaemia. Excess intake of fat, or calories in general, over a long period of time can lead to elevated levels of circulating triacylglycerol-rich lipoproteins (TRLs), especially very-low-density lipoprotein (VLDL) particles and remnants [2]. High levels and prolonged circulation of TRLs are considered atherosclerotic risk factors for cardiovascular disease [3].

The postprandial effect of different fatty acids may be ascribed to their variety in rate of oxidation, which in turn depends on chain length and degree of unsaturation. A comparative study on rats of the postprandial effects of different 18-carbon fatty acids indicated that ALA, more than stearic-, oleic-, and linoleic acid, promoted early use of lipids for oxidation, which spare the use of dietary carbohydrates [4]. A similar result was observed earlier in humans [5]. The potential health benefits of ALA for humans and the mechanisms involved are, however, still the subject of debate, especially the importance of the elongation and desaturation of ALA to eicosapentaenoic acid (EPA). The recent multicentre, double-blind randomized Alpha-Omega trial, showed that 1.9 g of ALA per day significantly increased the ALA and eicosapentaenoic acid (EPA) content of plasma cholesteryl ester [6]. The overall conclusion was that EPA + docosahexaenoic acid (DHA) and/or ALA supplementation at low doses did not reduce the occurrence of major cardiovascular events in patients with a previous myocardial infarction who were receiving good clinical care. There was an indication of a 27% reduction (P = 0.07) in cardiovascular events in women on ALA supplementation. Barceló-Cobijn and Murphy concluded that ALA is capable of providing and maintaining adequate amounts of DHA, especially in the liver and brain, provided that sufficient ALA (>1200 mg/day) is consumed [7]. Hodson et al. have published an extensive review on fatty acids as biomarkers [8]. It shows that ALA is present in all the measured pools (total lipids, TAG, NEFA, phospholipids in plasma and platelets, and adipose tissue), but in small amount since the dietary intake is low.

The lipid metabolism of men and pre-menopausal women differs. Women have lower levels of circulating TAGs and higher accumulation of fat subcutaneously, and in the gluteal and hip regions than men, who have a greater tendency to accumulate fat in the upper body and around the visceral organs (liver, kidneys, intestines, spleen). Men have an increased predisposition to mobilise FAs from visceral adipose tissue, but also have a higher FA uptake in these regions than women [1]. Men and women also secrete different amounts and sizes of TRLs from the liver [9]. Furthermore, women are more prone to store and/or convert alpha-linolenic acid (ALA, 18:3) to long-chain polyunsaturated FAs PUFAs rather than subject it to beta-oxidation [10].

We produced a new oil rich in ALA (ALA-rich oil), which has high contents of ALA and oleic acid, both with potential positive metabolic effects, by the enzymatic interesterification of linseed and rapeseed oil. We have previously investigated the postprandial lipid responses after consumption of the ALA-rich oil, olive oil and butter in men [11], and our main finding was that butter elicited the lowest increase in plasma (p)-TAG response, and the difference exceeded that which would be expected due to the presence of short- and medium-chain FAs in butter. Moreover, a significant increase in ALA was seen in total lipids, TAGs and NEFAs in plasma after the ALA-rich oil, as well as a sizeable recirculation of ALA into the NEFA pool.

Previous studies have shown that postprandial lipaemia differs between men and women [12,13]. We therefore investigated the postprandial plasma lipid responses in healthy pre-menopausal women after meals containing the new ALA-rich oil, butter, and olive oil. The FA composition in total lipids, TAGs and NEFAs in plasma was monitored for seven hours, and the 18:2 n-6/18:3 n-3 p-TAG ratios were calculated. The results are compared with the corresponding results previously obtained for men.

## Methods

### Study population

Nineteen healthy females, aged 25-50 years, were enrolled in this postprandial study. Recruitment advertisements were posted in the Biomedical centre news-letter at Lund University and at Skåne University Hospital. Subjects were enrolled after reading the written information, fulfilling the inclusion criteria and agreeing to participate. Their characteristics are given in Table 1. Habitual nutrient intake was assessed from food intake diaries during four days before each trial day (Table 1). The diaries and composition of the test breakfast were analysed using the Dietist XP program with the Swedish National Food Administration database from 2008. Dietary intakes were found to be close to the recommended Swedish dietary guidelines [14], and there was no difference regarding energy or fat intake on the day proceeding the three different test days. The exclusion criteria included pregnancy, lactation, dieting, pathological haematological parameters, high lipid and cholesterol levels, impaired kidney and liver function, current use of lipid-lowering or antihypertensive medication, milk-protein allergy or lactose intolerance. Oral contraceptives were not part of the exclusion criteria.

**Table 1:** Baseline plasma metabolite concentrations of the 19 female subjects (age (y) 34 ± 8, weight^1 ^(kg) 64 ± 10, BMI^1 ^(kg/m^2^) 23 ± 3 and waistline^1 ^(cm) 74 ± 8) and mean habitual nutrient intake

	ALA-rich oil Mean ± SD	Olive Oil Mean ± SD	Butter Mean ± SD
**p-TAG (mmol/L)**	0.83 ± 0.3	0.94 ± 0.4	0.83 ± 0.4
**p-NEFA (mmol/L)**	0.27 ± 0.1	0.34 ± 0.2	0.32 ± 0.2
**p-Cholesterol (mmol/L)**	4.5 ± 0.7	4.5 ± 0.8	4.6 ± 0.9
**LDL-C (mmol/L)**	2.5 ± 0.5	2.5 ± 0.6	2.5 ± 0.7
**HDL-C (mmol/L)**	1.6 ± 0.3	1.6 ± 0.3	1.6 ± 0.3
**Apo B/Apo A**	0.44 ± 0.1	0.46 ± 0.1	0.45 ± 0.1
**p-Glucose (mmol/L)**	4.9 ± 0.3	4.9 ± 0.3	4.8 ± 0.3
**p-Lutropin (IE/L)**	9.9 ± 14	7.9 ± 6	8.4 ± 6

**Dietary intake^2^**	**Mean ± SD**	**Mean ± SD**	**Mean ± SD**
**Energy (MJ)**	8.8 ± 2	8.7 ± 1	8.5 ± 2
**Protein (% of energy)**	15 ± 2	15 ± 2	15 ± 3
**Carbohydrate (% of energy)**	49 ± 6	49 ± 4	49 ± 6
**Total fat (% of energy)**	33 ± 5	35 ± 4	34 ± 6
**Saturated fat (% of energy)**	12 ± 3	12 ± 3	12 ± 3
**Mono-unsaturated fat (% of energy)**	11 ± 2	12 ± 3	11 ± 4
**Poly-unsaturated fat (% of energy)**	5 ± 1	5 ± 1	5 ± 1

No significant differences between trials (ANOVA, Bonferroni).

^1 ^Information obtained at the first visit.

^2^Analysed with Dietist XP, version 3.1, with the Swedish National Food Administration database (2008).

### Study design and test meal protocol

A single-blinded, randomized crossover study design was used. Each subject consumed three breakfasts containing 35 g of either ALA-rich oil, produced by enzymatic interesterification of cold-pressed linseed and rapeseed oil [11], organic extra virgin olive oil, or 42 g of butter. The FA compositions of the fats are presented in Table 2. The subjects were randomized into three groups, which either began with the ALA-rich oil, olive oil or butter meal. On the day preceding each test, subjects were advised to eat as usual, avoid alcohol, and fast overnight from 9 pm. The following morning between 7 and 7.30 am, a fasting venous blood sample was obtained. The test meal was served between 7.30 and 8 am. The breakfast consisted of semolina pudding in which the different fats had been mixed (Table 3). The taste was disguised by jam, and the subjects could, therefore, not identify which fat was being served. After breakfast, the subjects were not allowed to eat or drink anything but water until after the last blood sample had been taken. Blood samples were withdrawn at regular intervals during a period of 7 h after the test meals. Satiety was estimated subjectively, after breakfast (approx. 15 min) and after 1, 3, 5 and 7 h, using a visual analogue scale (VAS). The subjects were asked to rate their feelings of hunger, fullness and desire to eat on a 10 cm scale. The subjects were tested during the follicular phase in the menstrual period, hence there was a washout period of one month between the meals. The study protocol was reviewed and approved by the Regional Ethics Committee in Lund, Sweden (Reference No. 2009/41), and all participants gave their written informed consent.

**Table 2:** Fatty acid composition of the ALA-rich oil, the olive oil and the butter

Total FA composition	ALA-rich oil (mol %)	Olive oil (mol %)	Butter (mol %)
**FA 4:0 to 12:0**	0	0	22.9
**14:00**	0	0	12.5
**16:00**	4.7	12.1	28.7
**18:00**	1.8	2.8	10.4
**18:01**	38.5	69.1	19.7
**18:02**	19.5	13.9	1.7
**18:3**	35.5	2.1	0.6
**Others**	0	0	3.4

The 18:2 n-6/18:3 n-3 ratios were 0.5, 6.6 and 2.8 for the ALA-rich oil and olive oil and butter fat, respectively.

**Table 3:** Composition of the ALA-rich oil (876 g/mol), olive oil (875 g/mol) and butter (fat-part 734 g/mol) meals^1^

	Amount	Energy (KJ)	Fat (g)	Protein (g)	Carbohydrate (g)
**ALA-rich oil**	35 g	1277	35^2^	-	-
**Olive oil**	35 g	1277	35^2^	0.2	0.2
**Butter**	42 g	1265	35^3^	0.3	0.2
**Milk for cooking, 3% fat**	0.225 L	595	7.1	8.1	11.4
**Semolina (wheat kernel)**	20 g	291	0.3	2	14.2
**Rye bread, one slice**	38 g	408	1	2.9	17.8
**Smoked ham, 3% fat or**	9 g	44	0.3	2	0
**soft Cheese^4^, 3% fat**	9 g	43	0.3	1.6	0.3
**Blackberry jam**	32 g	262	0	0.1	14.8
**Milk, 1.5% fat**	0.2 L	410	3.1	7.2	10.3

**Total**		**3290**	**47**	**23**	**69**

^1 ^Data from proximate analysis of ingredients. Analysed with Dietist XP, version 3.1, with the Swedish National Food Administration database (2008).

^2 ^Equivalent to 0.04 mol

^3 ^Equivalent to 0.05 mol

^4^Vegetarian alternative (6 vegetarians)

### Blood sample collection and biochemical assays

The blood samples were collected from the forearm vein. Collection and biochemical assays were performed by the Phase One Unit and the Clinical Chemistry and Pharmacology laboratory at Skåne University Hospital, Lund, Sweden (http://www.analysforteckning.usil.se). Approximately 15 mL blood was collected on each occasion, and analysed to determine p-TAG, total plasma cholesterol, LDL-cholesterol (LDL-C), high-density lipoprotein-cholesterol (HDL-C), and apo-lipoprotein A and B (Apo A and Apo B). To ensure that all the subjects were healthy, haematological parameters, kidney and liver function parameters, the C-reactive protein value (CRP), plasma glucose, and plasma lutropin (p-LH) were measured in the fasting blood sample taken at the first visit. Plasma glucose, p-LH, and CRP were analysed on all occasions.

Approximately 2 mL of EDTA-plasma from each sample was saved for p-NEFA and FA composition analysis. Plasma NEFA concentration (n = 19) was analysed with an enzymatic colorimetric assay (Wako NEFA HR 2 test kit, Wako Chemicals GmbH, Neuss, Germany) on a BIO-RAD 550 microplate reader (BIO-RAD Laboratories Inc., CA, USA). The FA composition in total p-lipids, p-TAGs and p-NEFAs (n = 10) was analysed as described previously by Svensson et al. [11].

### Calculations

The concentration of TRL-cholesterol (TRL-C), which includes cholesterol in chylomicrons, VLDL and their remnants, was estimated by subtracting the values for LDL-C and HDL-C from the total plasma cholesterol. The increase in concentration in p-TAG (or TRL) at a time x h (p-Δ-TAG (or Δ-TRL)) was calculated by subtracting the concentration at time = 0 h with the concentration at time = x h (x = 1, 3, 5, 7 h). The ratio 18:2 n-6/18:3 n-3 in p-TAG was calculated by dividing the mol% linoleic acid by the mol% ALA.

### Statistics

The data were normally distributed. The incremental area under the curve (iAUC) and the AUC were calculated using the trapezoid rule. Statistical analysis of the data was carried out with repeated measurement analysis of variance (ANOVA). The test fats were the fixed factor. The Bonferroni post hoc test was carried out when significant differences were found between diets (P < 0.05). The paired t-test (n≥10) was used to determine whether there were any significant differences (P < 0.05) between values at different points in time. Results are presented as means ± SEMs, with the 95% CI. The Pearson correlation coefficient, r, was calculated (and linear regression was performed) to determine if there was any correlation between the various data. To test whether the correlations were significantly different from each other, linear regression was carried out, and the residual for each x-y pair was squared and tested with Friedman's test and Dunn's multiple comparison test. The satiety measurements were analysed with one-way ANOVA, the Kruskal-Wallis test and Dunn's multiple comparison test. All statistical analyses and calculations of the iAUC were performed with GraphPad Prism (version 5.00, GraphPad software, San Diego, CA, USA).

## Results

### Postprandial plasma triacylglycerols and non-esterified fatty acids

**Figure 1:**
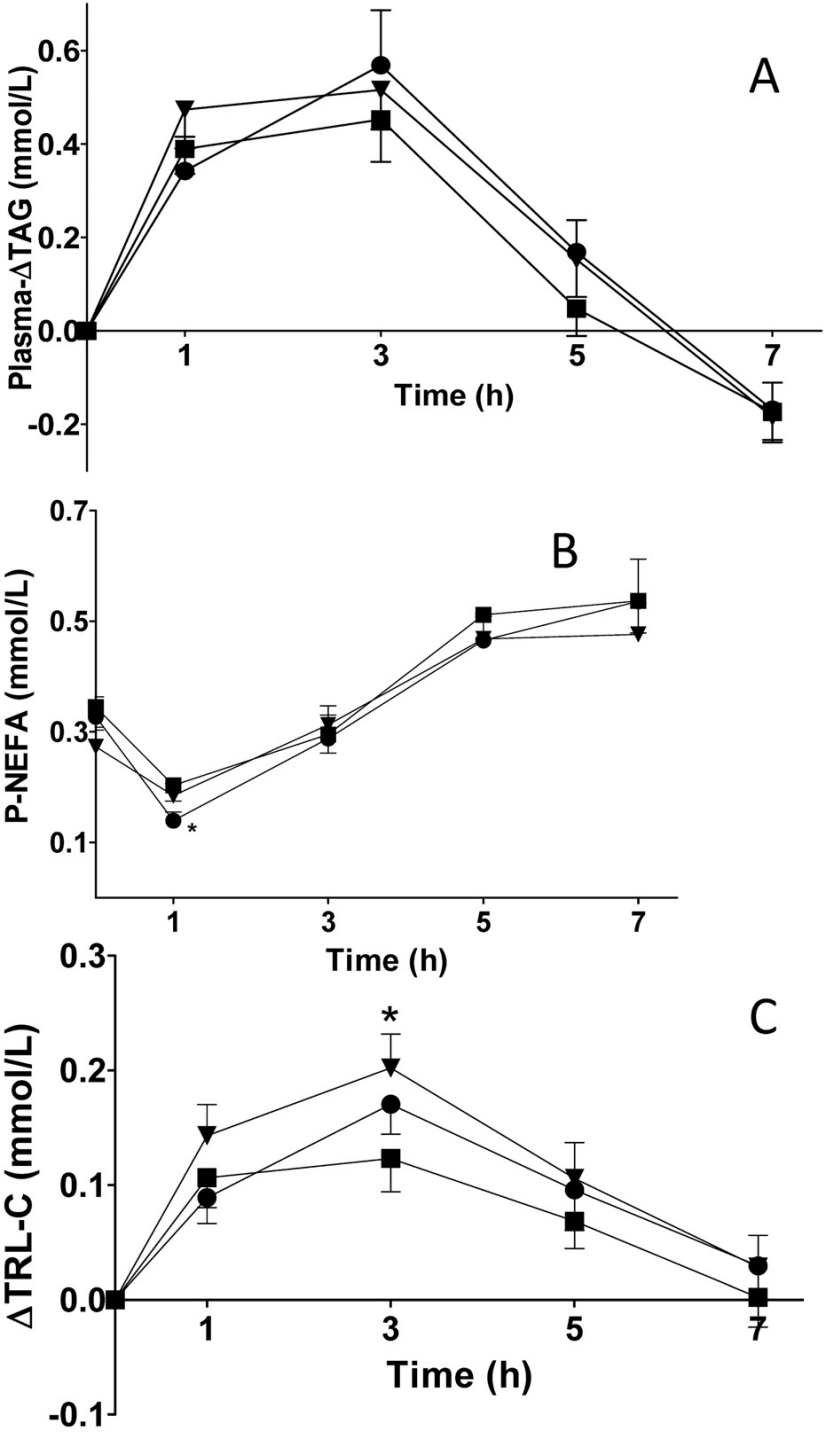
**Postprandial p-lipids in women (n = 19) after meals containing 35 g of fat from ALA-rich oil (-▼-), olive oil (-●-) and butter fat (-■-)**. **A**. Postprandial p-ΔTAG (mean ± SEM) versus time. **B**. Mean (± SEM) p-NEFA concentration versus time. The p-NEFA concentration following olive oil was lower than that following butter, 1 h after the meals (P < 0.05, ANOVA, Bonferroni). **C**. Δ-TRL-C is plotted against time. The Δ-TRL-C level 3 h after the butter meal was lower than that after the ALA-rich oil meal (P < 0.05, ANOVA, Bonferroni multiple comparison post hoc test).

The resulting postprandial p-ΔTAG and p-NEFA concentrations after the three different test meals are depicted in Figure 1. The butter meal induced lower iAUC (mmol/L·h) p-TAG response 1.7 (95% CI 1.3-2-2) than the ALA-rich oil 2.1 (95% CI 1.4-2.8) and olive oil 2.1 (95% CI 1.3-2.8) meals, however there was no statistically significant difference between meals (ANOVA) (Figure 1A.). The p-TAG AUCs after the three meals were not significantly different from each other (ANOVA, Bonferroni). The 95% CIs for the p-TAG AUCs were 7.7 (6.2-9.1 mmol/L·h) for the ALA-oil, 8.3 (6.6-10 mmol/L·h) for the olive oil and 7.3 (5.8-8.8 mmol/L·h) for the butter. Maximum levels of p-Δ-TAG were observed 3 h after all meals; the value for butter was lower than for ALA-rich oil and olive oil, but there were no statistically significant differences (ANOVA, Bonferroni).

P-NEFA concentrations showed an initial decline after one hour, followed by an increase between 1 and 7 h (Figure 1B.). There was no difference in the p-NEFA AUC between the meals (ANOVA). The 95% CIs for the p-NEFA AUCs were 2.4 (2.0-2.9 mmol/L·h) for the ALA-oil, 2.4 (1.9-2.8 mmol/L·h) for the olive oil and 2.6 (2.3-2.9 mmol/L·h) for the butter. The p-NEFA concentration at 7 h was significantly higher than at 0 h, after all meals (t-test, P-values: ALA-oil 0.0017; olive oil 0.0126; butter 0.0044), but no significant differences were found between the types of fat. The NEFA concentration was lower 1 h after the olive oil meal than 1 h after the butter meal (ANOVA, Bonferroni, P < 0.05). No such difference was found at subsequent points in time.

### Postprandial plasma cholesterol and lipoproteins

Total plasma cholesterol, LDL-C, HDL-C concentrations and the quotient between Apo B and A did not differ markedly between the different meals during the time period studied (ANOVA). LDL-C was higher at 7 h than at 0 h after the olive oil meal (t-test, P = 0.0297). The Apo B/Apo A ratio was lower 7 h after than directly after the ALA-rich oil meal (t-test, P = 0.0029). The Δ-TRL-C values are shown in Figure 1C. No significant difference was seen between the meals when comparing the iAUCs of Δ-TRL-C (ANOVA), but the value of Δ-TRL-C 3 h after the butter meal was lower than that 3 h after the ALA-rich oil meal (ANOVA, Bonferroni, P < 0.05).

### Fatty acid composition in plasma: Total lipids, triacylglycerols and non-esterified fatty acids

**Figure 2:**
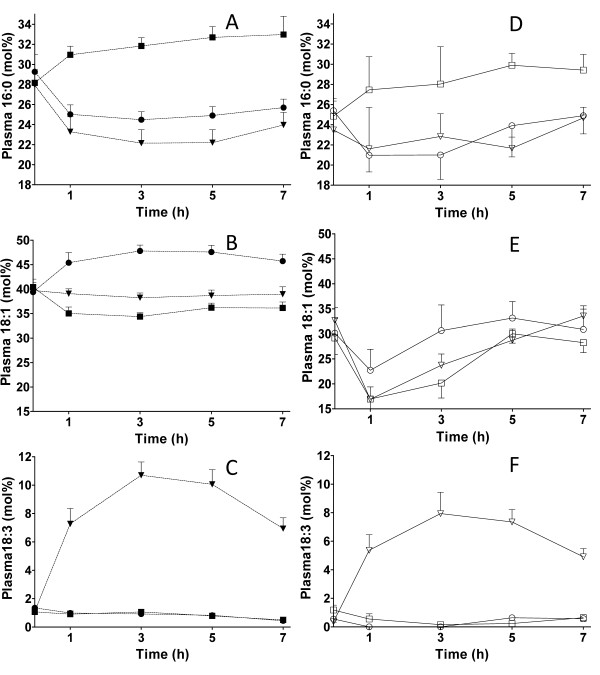
**Mean FA composition (mol% ± SEM) in p-TAG (n = 10): **A**: palmitic acid (16:0), **B**: oleic acid (18:1), **C**: ALA (18:3) and p-NEFA (n = 10):**D**: palmitic acid, **E **oleic acid, and **F **ALA, in women after meals containing 35 g fat from ALA-rich oil, olive oil or butter fat**. P-TAG: ALA-rich oil (--▼--), olive oil (--●--) butter (--■--). P-NEFA: ALA- rich oil (^-^∇^-^), olive oil (-○-) butter (-□-). The AUCs for the FAs that differed significantly between the meals are given in Table 4. Significance (P < 0.05, ANOVA and Bonferroni multiple comparison post hoc test).

The effects of the test fats on the FA compositions in total p-lipids, p-TAGs and p-NEFAs were analysed. Each meal had predominating FAs, i.e. palmitic acid in butter, oleic acid in olive oil and ALA in the ALA-rich oil. The mol% of these FAs in p-TAGs and p-NEFAs, are presented in Figure 2. The significant changes in FA composition in the different fractions, presented as AUC (mol%·h), after the test meals are given in Table 4. The FA composition in total p-lipids was changed after the ALA-rich oil and olive oil meals compared to fasting values. The percentage of palmitic acid in total p-lipids decreased between 0 and 7 h (t-test, P = 0.0005) after the olive oil meal, whereas the ALA-rich oil increased the percentage of ALA (t-test, P = 0.0036).

**Table 4:** The AUC (0-7 h) (mol%·h) for linolenic, oleic, linoleic and palmitic acid in total p-lipids, p-TAG and p-NEFA (n = 10), presented as means and 95% CI following the ALA-rich oil, butter and olive oil meals

	ALA-oil	Olive oil	Butter	ALA-oil	Olive oil	Butter
	**18:3 AUC (mol%·h)**			**18:1 AUC (mol%·h)**		

**Total p-lipids**	21	2.4^a^	2.3^a^	126^b^	142	119^b^
	(16-26)	(1.0-3.9)	(0.7-3.6)	(118-133)	(126-158)	(106-132)
**p-TAG**	60	6.1^c^	5.5^c^	271	324	243
	(47-73)	(4.4-7.8)	(2.5-8.6)	(258-284)	(306-342)	(223-263)
**p-NEFA**	42	2.3^d^	3.5^d^	175^e,f^	208^e^	167^f^
	(31-53)	(1.0-3.6)	(1.3-5.7)	(150-201)	(163-252)	(138-197)

	**16:0 AUC (mol%·h)**			**18:2 AUC (mol%·h)**		
**p-TAG**	162^g^	177^g^	217	108^h^	97^h^	72
	(142-181)	(165-188)	(195-240)	(96-120)	(86-108)	(63-81)

Only AUCs showing significant differences between the meals are shown (P < 0.05, ANOVA, Bonferroni multiple comparison post hoc test). The same superscript letters signify non-significant differences.

The FA composition in p-TAGs reflected the composition of the test fats (Figure 2A-C and Table 2). The most abundant FAs in each fat caused a significant change in the AUC corresponding to that specific FA (Table 4). The AUC for stearic acid was significantly higher after the butter meal than after the oil meals (ANOVA, Bonferroni P < 0.05) (data not shown). ALA peaked at 3 h after the ALA-rich oil meal (Figure 2C). The percentages of ALA after the ALA-rich oil meal (t-test, P < 0.0001) and the olive oil meal were higher after 7 h than the fasting level (t-test, P = 0.0032). This was also found for oleic acid after the olive oil meal (t-test, P = 0.0221). After the ALA-rich oil meal, the percent of palmitic acid was decreased after 7 h (t-test, P = 0.0127). The 18:2 n-6/18:3 n-3 ratio in p-TAG after the ALA-rich oil meal was 1.5 (95% CI 1.3-1.7) at 3 h and 2.4 (95% CI 2.1-2.7) at 7 h. The corresponding ratios after the olive oil meal were 13.8 (95% CI 12.0-15.5) and 16.9 (95% CI 11.7-22.1) and after butter: 9.0 (95% CI 6.8-11.3) and 11.6 (95% CI 6.5-17.6) at 3 and 7 h, respectively.

P-NEFAs were less influenced by the test fats than p-TAGs (Figure 2D to F., Tables 2 and 4). The fraction of ALA in p-NEFAs after the ALA-rich oil meal increased with time, and peaked at 5 h (Figure 2F). No decline was observed at 1 h, which means that the concentration is not concordant with the total NEFA curve (Figure 1B.). The only FA whose mol% was higher at 7 h than at 0 h was ALA after the ALA-rich oil meal (t-test, P < 0.0001).

The FA patterns in p-TAGs and p-NEFAs were different from each other. The AUC (mol%·h) for palmitic acid (after the olive oil and butter meals), oleic acid (all meals), and ALA (ALA-rich oil and olive oil meals) are all higher in p-TAGs and in p-NEFAs (ANOVA, Bonferroni, P < 0.05).

### Correlations

Elevated p-NEFA concentrations can increase the production of VLDLs and subsequently raise LDL-C levels [15], which may disturb the postprandial response of lipids [2]. After the olive oil meal, there was a positive correlation between the p-NEFA concentration after 3 h and both fasting and 3 h LDL-C (Pearson's r: olive oil 0.50 and 0.52, respectively). However, there was a lack of correlation between fasting p-NEFAs and fasting LDL-C. An increase in LDL-C has also been associated with increased chylomicron cholesterol concentrations [16], which can be seen during prolonged postprandial lipaemia [17]. In our study a positive correlation was found between iAUC TRL-C and LDL-C 0 h after olive oil intake (Pearson's r: 0.54). Increased fasting p-TAG levels, age, weight, and pre-test meal conditions are factors known to affect postprandial lipaemia [17-19]. However, no correlations were found between iAUC p-TAG and these parameters.

## Discussion

In this study we investigated the postprandial lipid response in healthy pre-menopausal women after the consumption of breakfasts enriched with 35 g of an ALA-rich oil, olive oil or butter fat. The FA composition in different plasma fractions was analysed, focusing on the most abundant FA in each test fat, e.g. ALA in the ALA-rich oil, palmitic acid in butter and oleic acid in olive oil.

Butter resulted in a lower postprandial iAUC p-TAG response than ALA-rich oil and olive oil, but there was no statistically significant difference between meals. The iAUC p-TAG responses after the ALA-rich oil and olive oil were approximately in the same magnitude. In our previous study on men, it was shown that the ALA-rich oil induced a lower, however not significant, lower response than olive oil [11]. In two separate studies, where the effects of meals containing walnuts, butter and olive oil were investigated, it was found that the total p-TAG response was greater following the olive oil meal than the walnut and butter meals [20,21]. Other researchers, who have also investigated the postprandial lipemic responses of ALA-containing meals and MUFA- rich meals, have found that there were no significant differences in p-TAG responses [22-24].

For the majority of the women the postprandial p-TAG concentrations were within the normal range (0.45-2.6 mmol/L) at all times after the test meals, which provided 0.75 g fat/kg body weight. The mean fat load in our previous study on men was 0.56 g fat/kg body weight, and their p-TAG responses were higher [11]. This shows that the clearance rate of p-TAG is more efficient in women than in men [9]. Pre-menopausal women are less sensitive to variations in dietary fat [12,25,26] and exhibit lower postprandial oxidative stress after fat intake than men [27], which can in part explain the lower prevalence of CVD in women in this age group. However, an elevated p-TAG level is a greater risk factor for CVD in women [1], which could mean that the highly efficient and regulated TAG metabolism in women is more sensitive to disturbances than in men. It has been suggested that the higher plasma lipid levels in men are the result of their greater capability to store fat in visceral adipose tissue and the greater catalytic activity of hormone-sensitive lipase in this tissue, which induces rapid FA delivery to the liver and higher production of VLDLs [1]. However, Magkos et al. showed that women had a higher VLDL-TAG secretion rate, a lower VLDL-Apo B_100 _secretion rate than men [9]. Moreover, it was also found that the mean residence times in the circulation for VLDL-TAG and VLDL-Apo B_100 _were shorter in women than men [9]. Steroid hormones such as oestrogen have been suggested to have anti-lipidaemic, antioxidant, and anti-inflammatory properties, but no effect was seen in a recent study on postprandial p-TAG response to the varying oestrogen level during the menstrual cycle [28]. On the other hand, when comparing the postprandial TAG-response in women, Gill et al found a lower response in the luteal phase than in the follicular phase. The postprandial TAG response in both phases were lower than the response in men [29]. In a review by Wang et al. it was concluded that estrogens and androgens may in part be responsible for the differences in lipid metabolism in men and women [30]. However, they argued that it is more likely also due to an intricate cooperation of a number of various hormone actions, such as by insulin, adipocytokines and different gene expressions [30]. The reasons and mechanisms behind the differences in lipid metabolism are still not completely elucidated. This confirms firstly, the importance of exercising postprandial investigations at the same time point in the menstrual cycle; secondly more studies are needed to understand the mechanisms of lipid metabolism in premenopausal women.

Our results suggest that the intake of ALA-rich oil has a denoted influence on the composition of p-NEFAs compared to the other meals. The p-NEFA is more influenced by endogenous sources, such as selective mobilization of FAs from adipose tissue, and de novo lipogenesis, than by the test fats from butter and olive oil. The iAUCs of palmitic and oleic acid in p-NEFAs were almost the same after the intake of the butter meal, despite the fact that butter contains 46% more palmitic than oleic acid. This could be partly explained by the fact that butter has the same amount of palmitic and oleic acid in the sn-1, 3 positions in TAG, hence the same amounts are susceptible to intestinal hydrolysis and partitioning to p-NEFA.

After the ALA-rich oil, ALA partitioned more to p-TAG than to NEFA. The total NEFA in plasma was at its lowest one hour after the meal (Figure 1B), while the ALA concentration was elevated (Figure 2F) at the expense of oleic acid (Figure 2E) and linoleic acid (data not shown). The high concentration and long circulation time of ALA in p-NEFA is indicative of recirculation of ALA from the adipose tissue and spillover NEFAs from the hydrolysis of newly formed VLDL-TAG [10,31]. ALA seems to be more important in other tissues than adipose, such as muscle and heart, where it can undergo beta-oxidation and become part of the carbon recycling process. According to Burdge and Calder [10], ALA is partitioned to beta-oxidation to a higher extent in men, whereas the conversion rate of ALA to longer PUFAs is higher in women.

The positive correlations between the p-NEFA concentration 3 h after the meal and LDL-C concentration both at fasting and at 3 h (after olive oil), and the lack of correlation between fasting NEFA and fasting LDL-C, observed in this study, are in line with the increasing belief that postprandial measurement of lipaemia is a more accurate determinant of atherogenic risk [32]. Women showed a significantly lower p-NEFA response (AUC) than men after the olive oil and butter meals [11], which can stem from the more efficient capture and re-esterification of FAs after hydrolysis of TRLs in the adipose tissue in women [31]. The total p-NEFA response did not differ between meals, but the p-NEFA concentration 1 h after the olive oil meal was lower than that 1 h after the butter meal. A probable cause of the higher p-NEFA concentration 1 h after the butter meal is the rapid release of short- and medium-chain FAs as plasma NEFA.

The FA distributions in p-TAG and p-NEFA were evaluated by calculating the increase in each FA (iAUC, mmol·h, results not shown). The p-TAG fraction reflected the FA composition of the fat ingested. After the ALA-rich oil meal, the iAUCs of FAs were in the order: oleic acid > ALA > palmitic acid, which corresponds to the composition of the ALA-rich oil (Table 2). The same pattern was observed for both olive oil and butter. Similar results were seen in a study by Mekki et al. in men aged 20-29 years, after the ingestion of meals containing 40 g of butter, olive oil, or sunflower oil [33], whereas another study on men and women aged 50-65 y showed no change in FA composition in p-TAGs as a result of the intake of a meal rich in mono-unsaturated FAs (48 g fat) [34].

The ALA-rich oil resulted in a lower 18:2 n-6/18:3 n-3 ratio in p-TAG than olive oil and butter, which persisted up to 7 h after the meal. Moreover, the ratio between saturated and unsaturated FAs in p-TAG was lower after the intake of the oils than after the butter meal. It has been argued that a balanced n-6/n-3 FA ratio in the diet is important [35,36]. ALA competes with linoleic acid (18:2) for the same desaturation and elongation enzymes. If ALA suppresses n-6 FA metabolism more than linoleic acid suppresses n-3 FA metabolism, as suggested by Holman [37], it is possible that an increase in ALA intake could decrease the level of n-6 FA metabolites such as arachidonic acid, and thus reduce the severity of inflammatory processes [7,36]. Since atherosclerosis and obesity are classified as low-grade inflammatory diseases [3], it could be beneficial to increase ALA consumption. In a recent study, it was shown that the consumption of food products from linseed-fed animals and linseed-enriched bread led to maintained n-3 levels in red blood cells in obese humans [35]. Furthermore, the ALA concentration in red blood cells was increased and the n-6/n-3 ratio increased in the experimental group (low PUFA/SFA and n-6/n-3 FA diet) compared with the control group (higher PUFA/SFA and n-6/n-3 diet). Geppert et al. found that women had a higher percentage of DHA and lower ARA/EPA and ARA/DHA ratios in platelet phospholipids than men, despite the fact that there was no difference in their diets or n-6 and n-3 FA intakes [38]. They hypothesized that the lower n-6/n-3 FA ratio may reduce platelet aggregation and vaso-occlusion. However, studies on ALA and inflammatory markers in relation to CVD have failed to show any conclusive correlations [39].

Subjective measures of hunger, fullness, and desire to eat using a VAS showed no difference in any of the parameters for the different meals. More objective measures could be obtained by the analysis of gut hormones related to hunger and satiety such as PYY and GLP-1, which are influenced by the fat content in meals and inhibit bowel movement (ileal break). Future studies should also include quantitative analysis of the distribution, size and amount of the TRL particles, Apo B-48, and Apo B-100, to more accurately evaluate the atherogenic risk. Investigations into whether the consumption of the new ALA-rich oil could affect markers of inflammation in subjects with chronic inflammatory diseases, such as obesity and atherogenesis, are warranted.

## Conclusions

No significant difference was found in the iAUC for p-TAG between the three meals investigated. The FA composition in p-TAGs strongly reflected the FAs present in the test fats, whereas p-NEFAs were predominantly influenced by endogenous sources. However, the ALA from the ALA-rich oil increased in all plasma lipid fractions. The ratio of saturated to unsaturated FAs in p-TAGs was reduced after the intake of the ALA-rich oil and the olive oil. The 18:2 n-6/18:3 n-3 ratio in p-TAG was lower after the ALA-rich oil meal than after the olive oil and butter meals 7 h after the meals. We conclude that future food products containing an ALA-rich oil can significantly affect plasma lipids and improve the n-6/n-3 FA ratio several hours postprandially, and thus form part of a healthy diet. Despite the general hormonal protection against detrimental plasma lipid levels in pre-menopausal women, the cumulative negative health effect of an unhealthy diet cannot be excluded. It is therefore important to investigate the effects of dietary lipids that can be implemented in women prior to menopause.

## Competing interests

The authors declare that they have no competing interests.

## Authors' contributions

All authors have read and approved the final manuscript. JS and LO were responsible for the design of the study, the performance, the data collection, the analysis of the results, and the writing of the manuscript. AR participated in the performance of the study, the data collection and the analysis of the results, and commented on the manuscript.
